# The Thought of Death in a Pandemic Era: Can Anxiety Determine the Nexus between the Accessibility, Availability and Use of Personal Protective Equipment (PPE) for COVID-19 and Work Behaviour among Aviation Workers?

**DOI:** 10.3390/healthcare10020215

**Published:** 2022-01-22

**Authors:** Edmund Nana Kwame Nkrumah, Suxia Liu, David Doe Fiergbor, Linda Serwah Akoto

**Affiliations:** School of Management, Jiangsu University, 301 Xuefu Road, Jingkou District, Zhenjiang 212013, China; nkrunak@gmail.com (E.N.K.N.); fdaviddoe@gmail.com (D.D.F.); Amalinda9@gmail.com (L.S.A.)

**Keywords:** death anxiety, COVID-19, pandemic, personal protective equipment (PPE), occupational health, work behaviors

## Abstract

Since the emergence of COVID-19, the aviation sector has been one of the numerous industries which have been affected the most. In this present paper, the thought of death among aviation workers as an indicator of anxiety at a time when the availability, access to, and use of Personal Protective Equipment (PPE) remains paramount to the survival of work in the line of duties and its influence on different work behaviors was assessed. The theoretical foundation of the study was built on the process efficiency theory, attentional interference theory, and the terror management theory (TMT), which focuses on both the psychological and emotional responses exhibited by people due to fear or worry about a specific situation. The study adopted an exploratory study design that incorporates a cross-sectional and self-reported survey among 646 frontline workers across 12 international airlines and the Ghana Airport Company Limited (GACL), Accra, Ghana using simple random sampling and convenient sampling techniques. After all the preliminary tests were performed, the path analysis estimated by Structural Equation Modelling (SEM) indicated that anxiety has a significant influence on workers’ stress-adaptive performance and task performance, but recorded no significant causal link with interpersonal performance. The findings indicated that all three proxies of employee work behaviours, which focus on both adaptive and task performance, were significantly related to workers’ access, availability, and use of PPE (APPE). The association between anxiety and APPE was also found to be significant. Bootstrapping mediation analysis shows that anxiety partially mediates the influence APPE has on both stress-adaptive performance and task performance, but did not show any mediating effect on the association between APPE and interpersonal performance. Among the three dimensions of death anxiety, both the fear of death (FDE) and death intrusion (DINT) indicated a significant partial mediating effect on the influence APPE has on all three multidimensional constructs of work behaviours. The findings literally prove that worrying about the fatality risk associated with COVID-19 is highly predictive.

## 1. Introduction

There is an increasing body of research that suggests that the thought and fear of death may be at the root of frequent mental health disorders or conditions, and it has been suggested that they must be addressed satisfactorily to achieve a long-term positive outcome. The emergence of SARS-CoV-2 in late 2019 remains a global health threat to the quality of life and has brought fear, worry, and the thought of death closer to most people than expected. As health workers and researchers continue to research for a vaccine for this deadly virus, what makes its control and cure quite challenging are the evolving variant strands. The virus is contagious through various transmission mediums: contact transmission (skin-to-skin contact with an infected person or by touching contaminated surfaces), respiratory transmission (coughing, sneezing, talking, singing, and laughing), droplets (resulting from an infected person talking, sneezing, or coughing), and airborne transmission through droplets which contain the pathogen have the potency to travel a longer distance from an infected person, and can also remain in the air for up to two hours [1]. Studies have established that migration and international travel remains the greatest medium by which the SARS-CoV-2 infection was exported and spread into different countries, and within a short time, SARS-CoV-2 became a global health concern [1].

Undebatably, the use of airplanes as a popular medium of international travel posed the risk of infection of the coronavirus to passengers, and possibly subsequently to aviation workers who interact with these passengers. It is common knowledge that the aviation industry constitutes a significant aspect of swift access to any country; hence, the emergence and spread of the COVID-19 virus across international borders ignited health concerns for aviation workers. As widely speculated, such workers in the process of administering their responsibilities interact with different passengers from all walks of life, and hence, are more likely to be exposed to the spread of the virus and subsequently face health complications in their line of duty [2].

Khan et al. [3] noted that on 1 March 2020, an indexed patient infected 15 other passengers on a 10 h flight from London, UK to Hanoi, Vietnam. The study of Pavli et al. [4] which was conducted on passengers on 18 international flights from 26 February to 9 March 2020, arriving at or departing from Greece, identified 21 indexed COVID-19 cases with four passengers and a crew member as victims of the infection. In a flight between Japan to Brazil in January 2021, the study of Fujino et al. [5] confirmed the presence of the coronavirus on four travelers. In most cases, especially in Africa, the coronavirus was first imported by cases that were detected at the airports. In March 2020, Ghana recorded its first two Sars-Cov2 infections which was an imported case from the United Kingdom (UK). The point is, international travel and airports, which represent an aspect of the aviation industry, have been one of the major conduits for the spread of the coronavirus. At a time when total restrictions cannot be placed on airport operations, it is imperative that the operations of the aviation sector will focus on protecting the health, safety, and quality of life of their workers. In Australia, a study among 810 participants revealed a significant positive correlation between death anxiety and anxious beliefs and behaviours related to COVID-19 [6]. Thus, in the wake of the coronavirus, where airplanes have been identified as “super spreaders” of the coronavirus, the availability and accessibility of PPEs for aviation workers are expected to be one of the basic mitigating measures for the risk of infection. More significantly, the mental, emotional, and psychological health concerns and performance of workers who facilitate the operations of this industry are also significant to its success in both the current pandemic and post-pandemic situation.

### 1.1. The Current Study

Beyond the loss of millions of lives due to coronavirus infections, the outbreak of the virus has also been a major source of mental health risk [7]. The fear of contraction of the virus and several conspiracy theories being propagated on social media are contributing factors to the heightened anxiety level among individuals. The speed at which the virus spreads and its preventive measures against the spread have been tipped to have implications on both mental health and individual well-being [8]. Thus, mental health-related factors, such as emotional and psychological stress, especially intrapersonal anxiety levels, have all been identified to be associated with the spread of the virus [9]. The high morbidity rate increased anxiety and fear [10]. “The constant fear of death may be keeping people anxious” [11]. Additionally, it is expected that workers’ perception of insufficient support from their organization increases their levels of anxiety and fear [12]. Amidst these, a shortage or lack of PPEs has been identified as a significant contributory factor feeding into the level of anxiety and fear among workers [13].

It is worth noting that anxiety among employees has undeniably been a major cause of loss of concentration and increased worry and under-productivity. According to Eysenck and Calvo [14], anxiety obstructs performance and is a threat that may trigger behavioural reactions to negatively affect workers’ cognitive abilities [15]. Precisely, performance disruption is proportional to the magnitude of anxiety being experienced by an individual. Some studies have also shown that unacceptable behaviours mostly exhibited at the workplace that are likely to hinder work performance can be linked to anxiety [16]. In this study, anxiety was adopted as a mediating variable that is likely to determine the relationship between access to PPEs and performance among aviation workers. Just like frontline health workers and their dire need for PPEs, aviation workers also need the same level of attention, as they mostly remain the first point of contact with travelers who may be carrying the virus [17]. Aviation workers are more or less like frontline health workers, yet enough attention has not been given to them.

In the quest to establish a strong conceptual framework that addresses the issue of anxiety as a determinant of employee work behaviours, the study diverges from other studies by focusing on work behaviour variable indicators that are reflective of the current work situation which is highly related to the workplace responsibilities of aviation workers. Thus, more emphasis was given to employees’ level of work adaptability by assessing their stress-adaptive performance and interpersonal performance, while also evaluating their task performance. Thus, the study defined a work behaviour proxy that captures both the cognitive and emotional construct of workers. Needless to say, in an era of a pandemic, the need for the psychological and emotional orientation of workers to adapt to variant situations and still perform a work-related task is of the essence. As a positive responsive measure to uncertainty, versatility, adaptability, and tolerance are important features that workers must garner for effectiveness and efficiency in the working environment [18]. It is worth mentioning that, irrespective of the abundant studies conducted over the past 2 years relating to COVID-19, none of the studies have assessed the objectives this study seeks to explore. The context of the study’s objectives will therefore provide valuable information for the government, private organizations, and policymakers on the need to control and manage anxiety and employee behaviour towards work in future health-related emergency crises.

### 1.2. Conceptual Framework and Research Purpose

According to the process efficiency theory, anxiety “pre-empts some of the processing and storage resources of the working memory system” that hampers performance [14]. Anxiety, avoidance motivation, and the arousal theory postulate that anxiety (worry) is “equated with avoidance motivation, which produces a subsequent decrease in resources allocated to the task (on-task effort)” [19]. On worry and the attentional interference theory, Liebert and Morris [20] established that anxiety comprises worry and emotionality, and opined that anxiety (worry) is an important factor that decreases the employee’s performance. On worker behaviour, the terror-management theory (TMT) framework can be attributable to both the psychological and emotional responses exhibited by workers towards the outbreak of the coronavirus. Thus, the TMT posits that the fear of death is a major driver of human behaviour [21]. By incorporating these theories into the study, it can be assumed that access to PPEs, which remain a significant barrier between humans and the virus in such an exposed work environment may cause a high level of anxiety (fear and worry) and consequently influence employees’ work behaviour.

The study of Plaisier et al. [22] established that workers with anxiety have a six times higher risk of experiencing problems related to their performance at work as compared to other workers. A large study involving 810 Australians that specifically explored fears of death in the context of the pandemic revealed that there is a significant positive correlation between death anxiety, beliefs, and behaviours related to COVID-19 [6]. Adapting data from OECD countries, the findings of Leontaridi and Ward [23] also reveal a positive significant association between self-reported work-related stress and anxiety, and individual intentions for quitting and absence from work. Consistent with the Karasek [24] model, the findings of Wood [25] suggested that there exist greater anxiety in more demanding jobs with lower levels of employee control. Unarguably, anxiety has become more pertinent than ever in the context of the current pandemic, as some findings have suggested that fears of death through the virus exist.

Consequently, the quest to decrease the danger of job exposure to a contagion means that the use of PPE is paramount [26]. A recent study conducted in Hubei Province, China, identified the use of PPE as one of the physical and psychological challenges experienced by the physicians while responding to COVID-19 [27]. The study of Dejanovic and Heleta [17] further suggested that to ensure the safety of aviation employees, workers should use personal protective equipment and must be trained to adhere to health and safety measures. More importantly, access or availability of PPEs is very significant in mitigating or controlling the risk of anxiety among workers, as it is found to lessen the fear that marks opinions on individual risks and adherence to preventive safety measures [28]. Few studies have advanced the idea that death anxiety, for instance, may be a driving force for quite severe psychological distress during the pandemic.

As cited by the WHO, the severe and mounting disruption in the global supply of PPEs instigated by an increase in demand, panic buying, hoarding, and misuse placed a lot of lives at risk. Just like healthcare workers who rely on personal PPEs to protect themselves and their patients from being infected and infecting others, aviation workers, specifically the frontline staff, also need PPEs as their first defensive wall. The potential consequences and the related health risk for failing to provide adequate PPEs are clear. Thus, if workers at international airports cannot be protected, then the public is equally unsafe. This analogy may as well feed into causing a high level of anxiety among these unprotected workers and the public. Certainly, new related findings across the world have cited COVID-19 as a significant determinant of mental health and increases in psychological issues [29,30,31,32], with most studies referring to anxiety and depression as a major concern due to the several restrictions and prevention protocols established by the World Health Organization (WHO), Geneva, Switzerland. Schimmenti et al. [33] conceptualized the fear of COVID-19 as comprising four domains (bodily, interpersonal, behavioural, and cognitive) with each domain having a distinct aspect. Consequently, the inadequate supply of PPEs has also been named as a potential threat to increases in depression and anxiety, specifically among most frontline health workers.

Building the study’s objectives on these empirical evidences, the study seeks to examine the impact of anxiety among aviation workers’ work behaviors. In the context of this study, work behaviors consist of employees’ task performance, stress-adaptive performance, and interpersonal performance; therefore, the influence of anxiety on these three proxies of work behaviors were also evaluated. As PPEs remain a vital pillar in the fight against the pandemic, the study further proposes to understand the influence of the availability and access to PPEs on the work behaviors of aviation workers. Additionally, in this era of different waves of the coronavirus, anxiety is expected to occur among aviation workers if there is an inadequate supply of PPEs, or there is a lack of access to or training on the proper use of PPEs. The occurrence of this events should consequently dictate workers’ approach towards work; hence, anxiety is expected to be a suitable mediator in the causal link between the availability and access to PPEs and employees’ work behavior (i.e., stress-adaptive performance, interpersonal performance, and task performance).

**Figure 1:**
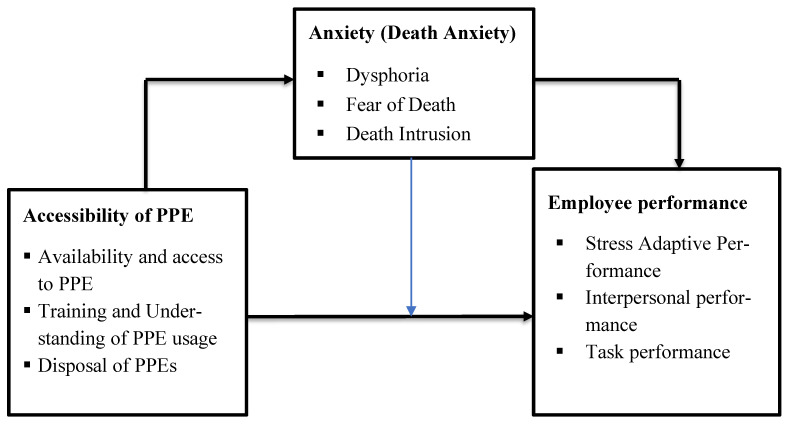
Conceptual framework.

The conceptual framework based on the research purpose and empirical evidence is presented in Figure 1 below:

## 2. Methods

### 2.1. Study Design and Sampling Technique

The study adopted an exploratory study design that incorporates a cross-sectional and self-reported survey administered to employees within the aviation sector of Ghana. Ghana currently has only one major international airport located in its capital, Accra. Kotoka International Airport (KIA), Accra, Ghana, as it is popularly known, inhabits over 35 combined international and local airlines. Apart from flight and aviation management, the airport is also a place to shop and dine, and hence has places like lounges, restaurants and mini-malls. The managers and supervisors of all standardized operations in KIA are the Ghana Airport Company Limited (GACL). During this pandemic, the GACL has played a tremendous role in ensuring that safety, health, and all COVID-19 protocols were observed by all players within the confinement of the airport space.

In this study, the frontline employees of 12 international airlines operating in KIA and the frontline managers and supervisors of the GACL constituted the sample framework. Frontline workers in this study constitute employees whose work responsibilities position them into direct contact with travelers and customers. Simple random sampling and convenient sampling techniques were utilized to select respondents due to the numerous restrictions and strict COVID-19 protocols at the airport. These sampling techniques were preferred because data were solicited from frontline workers who are readily available, accessible, and willing to participate in the study at the specific data collection period. A total of 729 respondents constituted the study, however, data analysis was based on only 646, due to several errors in responses and incomplete questionnaires. Most responses were solicited through e-mails, the use of online survey tools, and telephone calls.

In compliance with acceptable standards and research ethics, permission was sought from appropriate quarters. Respondents were well-oriented and willingly allowed to respond to questions at their own convenient and appropriate time. No respondent in the entire study was oppressed, punished, or given any form of financial reward to accept or deny partaking in the study.

### 2.2. Definition of Variables

#### 2.2.1. Accessibility, Availability and Use of Personal Protective Equipment (PPEs)

In the context of this study, personal protective equipment (PPE) refers to the accessibility, availability, and use of special health equipment worn to reduce the chance of touching, being exposed to, and the spreading of germs, infections, and viruses. In the era of this pandemic, the use of PPEs is therefore expected to create a barrier between a person and the spread of the coronavirus. The dimension of PPEs in this study therefore focuses on the availability of and access to PPEs, training, and understanding the use of PPEs and the disposal of PPEs. The types of PPEs are confined to include surgical gloves, surgical and respiratory masks, face shields, protective goggles, disposable gowns and disposable shoe covers.

#### 2.2.2. Anxiety

Literally, anxiety is an unpleasant state of mental uneasiness, nervousness, apprehension, and obsession or concern about some uncertain event. Bearing in mind the behavioral costs, the context of this study, and the nature of anxiety in a pandemic era, this study focuses on the concept of “death anxiety” as the major parameter. This concept refers to the state in which a person experiences physical symptoms of being upset, nervous, and have feelings of dread, worry, and fear related to one’s own death and dying through an imagined threat to one’s existence [34]. The dimension and context of this definition fall on the physiological nervous reactivity of people, recurrent thoughts about death-related events, the feeling of worry and fear about death, and the avoidance of thoughts and events related to death.

#### 2.2.3. Work Behaviors

The concept of work behavior is contextually broad and highly subjective. However, it is paramount for every organization to understand why people behave the way they do. In this study, work behavior basically refers to the constructive and productive behavior of employees at the workplace. The concept of work behavior as adopted in this study is therefore synonymous with employees’ work performance. The work behavior construct was therefore confined to assessing stress-adaptive performance, interpersonal performance, and the task performance of employees.

Stress-adaptive performance was identified by Pulakos et al. [35] as one of the most significant performance dimensions in any working environment. They argued that employees must not panic, but rather continue to always make good decisions irrespective of their level of stress caused by changes in the work environment.

Interpersonal performance is the aspect of behaviors that include demonstrating interpersonal flexibility, adjusting their interpersonal style to achieve a goal, and adapting to work effectively with a new team or co-workers, or fulfill customer needs [36]. Pulakos et al. [35] believed that a good interpersonal relationship improves the working relationship and enhances performance.

Task performance, on the other hand, literally refers to the degree of effectiveness of work performance that feeds into the organizational goals and objectives [37]. Campbell [38] explained task performance as the ability of workers to substantively perform their core mandates as expected by the organization. In this study, employees’ task performance describes the performance of job responsibilities devoid of mistakes, handling of job demands, and always making the right decisions [39].

### 2.3. Measures

All the scales adopted for this study went through pilot testing, and minor modifications were made to fit the context of the study; hence, only scales that met the reliability test criterion through exploratory factor analysis were used for further analysis. Ambiguous and double-barreled questions were avoided for precision and clarity.

The survey measuring accessibility to PPEs was borrowed from the English version of a structured questionnaire developed by the University of Bologna (Bologna, Italy) and Harvard University, Cambridge, MA, USA, as used in the study of Savoia et al. [40] in addressing concerns as to PPEs among Italian physicians. The context of the survey embodies the access to and adequate availability of different types of PPE, training and knowledge on the use of PPEs, and the appropriate disposal of PPEs.

Anxiety was measured using three proxies adopted from the updated Scale of Death Anxiety (SDA) developed by Cai et al. [34]. This is a multidimensional construct that combines all other SDAs and assesses an individual’s somatic, cognitive, emotional, and behavioural reactions from asymptomatic perspectives. The dimensions for measuring anxiety, therefore, include assessing respondents’ perceptions on dysphoria, which measures a state of feeling emotional and mental discomfort; fear of death, which measures the reoccurring thoughts about death and dying due to COVID-19 exposure; and finally, death intrusion, which measures the feeling of thinking about one’s own death due to the worry and fear of COVID-19.

Concerning work behaviours, the adaptive performance construct as developed by Pulakos et al. [18] was adopted. The focus of this variable is to clearly address employees’ level of adaptability to changes in the work environment. The dimensions for the adaptive performance construct, therefore, include interpersonal adaptive performance, as well as stress-adaptive performance [41]. On the other hand, the task performance scale was adopted from the study of Abramis [39], and it focuses on measuring a worker’s proficiency in job skills, job knowledge, work quantity, and work quality.

All measurement scales were assessed on a 5-point Likert scale ranging from 1–5, where 1 represents “totally disagree” and 5 represents “totally agree” as a response to the items.

### 2.4. Data Analysis Procedure

Structural equation modelling (SEM) was used as the main method of data analysis. Specifically, the second-order factor model method was preferred. This method produces robust inferences relative to the traditional regression model. Descriptive and reliability analysis was initially performed to ascertain the means, standard deviation, and Cronbach’s alpha of all scales. The Kaiser–Meyer–Olkin Measure of Sampling Adequacy (KMO-MSA) and Bartlett’s Test of Sphericity (BTS) was also estimated. Consistent with prior works of Mardani et al. [42], the study set a strict level of significance for the regression coefficients at (95%) for each latent variable, and accordingly evaluated the reliability, validity, and internal consistency of each latent variable. Fit indices were further used as an indication to ascertain whether the model is acceptable or otherwise. The procedures of Baron and Kenny’s [43] bootstrapping method, which assesses the indirect effect of the mediating variable, were followed to estimate the mediating effect. Statistical Product and Service Solutions (SPSS) version 26.0 (IBM, Armonk, NY, USA) was used for data-coding, descriptive, reliability, and validity analysis, while the path estimates and confirmatory factor analysis was estimated through the use of version 25.0 of Analysis of a Moment Structures (AMOS).

## 3. Results

### 3.1. Description of Respondents

The majority of the respondents recruited for the study were frontline workers who mostly engaged with customers frequently in their line of work. The demographic statistics of respondents are provided in Table 1 below:

### 3.2. Descriptive and Reliability Analysis

The results as presented in Table 2 below indicate that the Kaiser-Meyer-Olkin Measure of Sampling Adequacy (KMO-MSA) of the scales were all above the 0.600 thresholds. Additionally, Bartlett’s Test of Sphericity (BTS) for the scales were all significant. The reliability statistics also indicated a Cronbach’s α greater than the threshold of 0.70 for all constructs. Both the skewness and kurtosis results also met the statistical assumptions for a normal distribution. According to Brown [44], acceptable values of skewness fall between −3 and +3, and kurtosis is appropriate from a range of −10 to +10 when utilizing.

### 3.3. Convergence Validity

The results for the average variance extracted (AVE) and composite reliabilities (CR) for all constructs, as presented in Table 3, met all the statistical conditions of a convergent validity test. Thus, both the AVE and CR of all constructs were above the threshold of 0.5 and 0.7, respectively.

### 3.4. Discriminant Validity

The findings as indicated in Table 4 below show that the scales are distinct and unrelated, as the square root of AVE for each scale was higher than their correlation with other scales. The results, therefore, satisfy the assumption that the items are unique and do not discriminate against their measurement construct [45].

### 3.5. Goodness-of-Fit Index and Structural Equation Model

The results of the fit indices were used to establish whether the overall model as conceptualized using the structural equation modelling is acceptable. The findings suggest all the model fit indices as indicated in Table 5 below satisfy the statistical criteria for the structural equation model.

**Figure 2:**
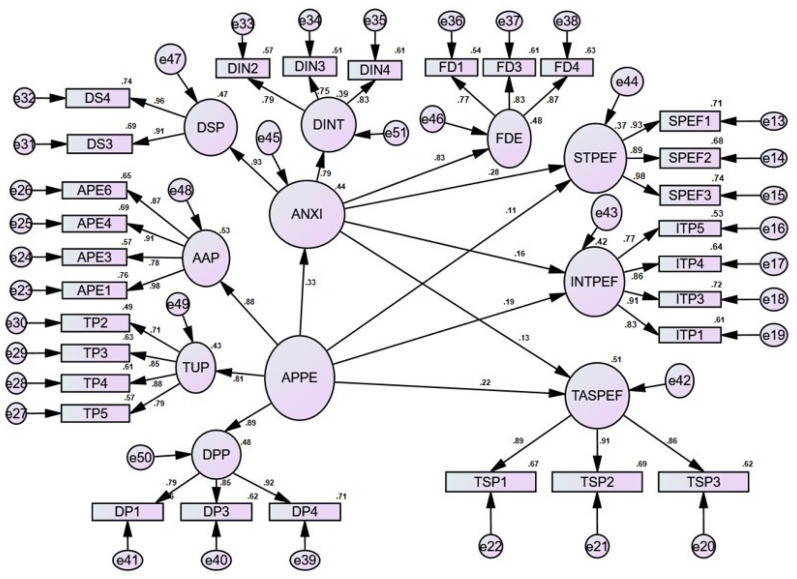
Structural equation model.

Further, the results for the Structural Equation Model are displayed in Figure 2 below:

### 3.6. Path Analysis

The results of the path analysis as estimated by SEM using the *p*-values of the path estimates, tested at a 95% confidence level in explaining the relationship between anxiety (ANXI) and work behaviours indicated 0.289 between ANXI and stress-adaptive performance (STPEF), 0.165 between ANXI and interpersonal performance (INTPEF), and 0.137 between ANXI and task performance (TASPEF). The path estimates clearly show that ANXI significantly determines both STPEF and TASPEF, but showed no significant influence on INTPEF. Based on the estimates, ANXI indicated a stronger significant effect on STPEF than TASPEF.

**Figure 3:**
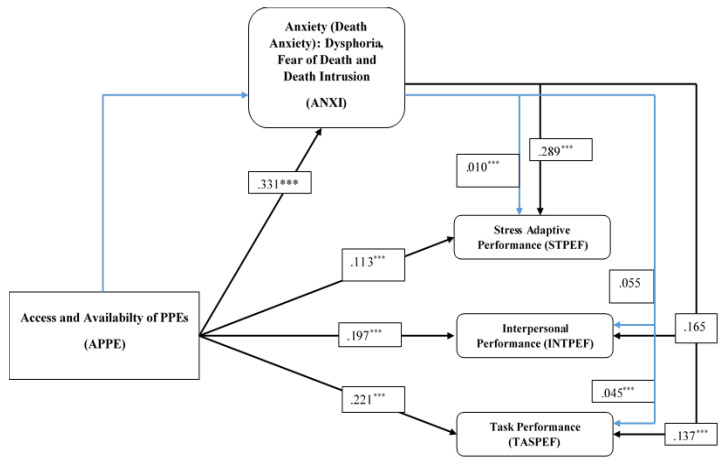
**Path analysis.** *** Significant at 95%, blue arrows signify mediation.

The paths explaining the causal link between the availability, access to, and use of PPEs (APPE) and all three proxies of work behaviours were significant. Thus, the path explaining the relationship between APPE and STPEF indicated a significant effect of 0.113, and APPE and INTPEF recorded 0.197 and 0.221 for the causal link between APPE and TASPEF. These results also show that APPE has the highest influence on TASPEF than INTPEF and STPEF, while the lowest causal link was recorded between APPE and STPEF. The basic analogy of these results is that the availability, access to, and use of PPEs significantly determine all aspects of work behaviours among aviation workers. Not least, the causal link between APPE and ANXI indicated a significant effect of 0.331. This means that a unit increase in APPE will determine workers’ anxiety levels by 0.331. The results for the path estimates are presented in Table 6 below:

The results for the path estimates are further illustrated in Figure 3 below:

### 3.7. Mediation Analysis

Fundamentally, the significance of the mediation model is to identify and describe the channel or process that underlies the observed association between the exogenous and endogenous variables. The study examines the mediation effect of anxiety on the causal relations between access and availability to PPEs and work behaviours by estimating the significance of the indirect effects of the mediating variable using the bootstrapping method. The results of the mediation effect indicate that anxiety (ANXI) partially mediates the causal relationship between access and availability of PPEs (APPE) and the stress-adaptive performance (STPEF) of workers. The indirect effect also indicates that ANXI partially mediates the causal association between APPE and task performance (TASPEF), but recorded no mediation influence on the causal link between APPE and the interpersonal personal performance (INTPEF) of workers.

To clearly define the significance of the mediation effect of anxiety, the study further estimated the mediation effect of each component of the anxiety constructs. Understanding this mediation effect is expected to address specific areas of anxiety and their determinant effect on employees’ work behavior. The findings suggested that both the fear of death (FDE) and death intrusion (DINT) partially mediates the causal association between access and availability of PPEs (APPE) and all three proxies of work behaviours (i.e., stress-adaptive performance, interpersonal performance, and task performance). On the other hand, dysphoria (DSP) partially mediates the influence of APPE on both stress-adaptive performance and task performance, but showed no significant effect on the causal relationship between APPE and interpersonal performance. The results literally mean that FDE and DINT, as a form of anxiety, comparatively hold the highest determinant of work behaviour among employees. The results of the mediation analysis are presented in Table 7 below.

## 4. Discussion

In this present paper, the thought of death among aviation workers at a time when the access and use of PPEs remain paramount to their survival in their line of duties and its influence on different work behaviours were explored. The theoretical foundation of the study is built on the process efficiency theory, attentional interference theory, and the Terror Management Theory (TMT), which focuses on both the psychological and emotional responses exhibited by people due to fear or worry about a specific situation [14,20,21]. The objectives for the study were purposely carved to explain the impact of access, use, and availability of PPEs (APPE) on death anxiety and three different proxies of work behaviour that focus on the task and adaptive performance of employees. The concept of death anxiety was explored from three dimensions (i.e., dysphoria, fear of death, and death intrusion) to ascertain its effect on the causal relationship between APPE and the multidimensional construct of employees’ behaviours.

After all the preliminary tests were performed, the findings of the study as estimated by the path analysis and structural equation modelling indicated that anxiety has a significant influence on workers’ stress-adaptive performance and task performance, but recorded no significant causal link with interpersonal performance. In the context of this study, the findings indicate that death anxiety is a significant determinant of workers’ ability to manage the stress associated with the rapid and unpredictable nature of change in the working conditions at the workplace, as well as the eagerness to accomplish their work goals in a timely manner and such that they are devoid of mistakes. As Pulakos et al. [35] opined, employees must not panic, but rather, continue to always make good decisions irrespective of their level of stress caused by changes in the work environment. As established by Plaisier et al. [22], compared to other workers, employees with anxiety have a six times higher risk of experiencing problems related to their performance. This finding does not only intensify the findings of previous studies, but also alludes to the proposition of the TMT, as anxiety has been proven to significantly determine human behaviour at the workplace. Some other studies have even highlighted a significant association between self-reported death anxiety and disorders among humans, such as post-traumatic stress disorder [46], depression [47], and lifetime mental health diagnoses [48].

Although anxiety has been proven by several studies to be a significant determinant of human behaviour, one may also agree that the concept of death in the minds of people due to the fear of contracting the virus, especially at a workplace that has been proven to be one of the highest sources of importing the virus, remain extremely relevant. Apart from other work-related issues that are likely to cause anxiety among workers, the thought and fear of death in an era of a pandemic is expected to increase the level of anxiety and cause severe cognitive distortion among employees. The theoretical position of Humphrey and Revelle [19]; Sarason [49], for instance, argued that anxiety is a major cause of worry, and worry mostly impairs task performance with high attentional or short-term memory demands. Additionally, the processing efficiency theory highlighted that anxiety causes a reduction in the storage and processing capacity of the working memory system available for a concurrent task, and hence, may impede work performance. Be that as it may, the relationship between anxiety and work behaviors as established in this study is not straightforward, as the findings suggest that anxiety did not significantly influence all three proxies of work behaviors.

In relation to the influence of the availability, and the use and access to PPEs (APPE) on employees’ work behaviours, the findings indicate that APPE has a significant casual relation with task performance, stress-adaptive performance, and interpersonal performance of employees. Thus, all three proxies of employee work behaviours, which focus on both adaptive and task performance, were significantly related to APPE, an indication that APPE remains an important resource that determines all aspects of workers’ behavior in the aviation sector. The relationship between APPE and task performance, however, recorded the highest significant relationship, while APPE and stress-adaptive performance recorded the lowest. This result is somewhat unsurprising; after all, in the wake of the coronavirus, where the airports have been identified as “super-spreaders” of infections, the availability and accessibility of PPEs remain the first, and perhaps the only defensive and protection mechanism for all. The World Health Organization (WHO), Geneva, Switzerland has highlighted and reiterated this over time since the emergence of the virus. During the World Day for Safety and Health at Work, the WHO called for an integrated approach between the governments, employers, and workers’ organizations, including the global community to take urgent measures to strengthen countries’ capacities to protect the safety of workers and to provide them with occupational health services [50]. Thus, one may agree that creating a safe workplace for workers in an era of the pandemic includes the continuous supply of PPEs, and the provision of adequate training and their suitable use is critical to protect the safety and health of workers and also improve their work behaviour. This assertion corroborates with the findings of Dejanovic and Heleta [17], who suggested that aviation workers should be provided with the necessary PPEs and must be trained to adhere to health and safety measures at the workplace.

Again, the association between anxiety and access, availability, and use of PPEs (APPEs) as estimated by the path analysis was also found to be significant. As indicated by Duan and Zhu [29]; Gallagher et al. [30]; Wang et al. [31]; and Xiao [32], the emergence of COVID-19 has significantly caused mental health problems among people, and the rise in anxiety has been named as a major concern; however, the access and availability of PPEs have proven effective in mitigating or controlling the risk of anxiety, as it is found to lessen worry and fear among people [28]. The study of Pérez-Mengual et al. [51], for instance, reported that women displayed higher scores on anxiety and fear of personal death, while in men, the fear of personal death mediated the relationship between neuroticism and anxiety. In the context of this study, where anxiety is defined in the confines of death anxiety, it is right to assume that the inevitability and unpredictable nature of death makes people feel a sense of horror, and this fear of death is a fundamental source of anxiety, hence its significant association with APPE [52]. The mismanagement of this kind of anxiety in this period where different waves of the COVID-19 pandemic are emerging may consequently aggravate symptoms of mental disorders [53,54]; hence, workers can only feel safe, emotionally stable, and experience a low level of anxiety if they have easy and adequate access to all needed PPEs for their work. Most studies have found a strong and consistent relationship between access to PPEs and risk of COVID-19 exposure across multiple countries [55,56,57], while other studies have also supported the importance of PPE in reducing worry and fear related to the transmission of COVID-19 [58,59].

Lastly, the findings related to the mediation effect of anxiety in the causal link between APPE and all three multidimensional constructs of work behaviour indicated that anxiety partially mediates the influence APPE has on both stress-adaptive performance and task performance, but did not show any mediating effect on the association between APPE and interpersonal performance. Among the three dimensions of death anxiety, both the fear of death (FDE) and death intrusion (DINT) indicated a significant partial mediating effect on the influence APPE has on all three multidimensional constructs of work behaviours. Statistically, this also means that FDE and DINT are significant determinants of task performance, stress-adaptive performance, and interpersonal performance. Unlike FDE and DINT, dysphoria (DSP) indicated no mediation effect on the relationship between APPE and interpersonal performance, but partially mediated the influence APPE has on both task performance and stress-adaptive performance. Be that as it may, the findings show that, to a large extent, all three-dimensional constructs of death anxiety are significant mediators in the relationship between APPE and employees’ work behaviour. Clearly, even with the availability, access, and use of PPEs by aviation workers, the existence of anxiety determines both their adaptive and task performance at the workplace. As indicated by Iverach, Menzies and Menzies [60], the existence of death anxiety remains a transdiagnostic construct that has contributed to the development and maintenance of numerous mental health disorders among employees, while the reminder that death is nearer or closer has been shown to increase social avoidance among participants high in social anxiety [54].

The findings of this current study corroborate and confirm the findings of Ahorsu et al. [10], whose evaluation of the psychometric properties relating to the Fear of COVID-19 Scale indicated that the item, ‘I am afraid of losing my life because of COVID-19’ recorded the highest factor-loading. This literally proves that a person’s worries about the fatality risk associated with COVID-19 is highly predictive. Some studies have also shown that estimates of fatality associated with COVID-19 have shown to significantly determine psychological distress, levels of stress, and depression, which are all predictors of behaviour among people [31]. It is important to iterate that, despite the proliferation of research related to the use of personal protective equipment (PPE) as safety and a preliminary shield in this era of COVID-19, no study has currently highlighted its association with anxiety, specifically in the context in which this study was defined.

## 5. Conclusions

The thought of death can potentially create a sense of powerlessness, loneliness, and worthlessness, and among some individuals, it may even undermine their experience of happiness or peace and how they relate with people, or even their performance at work. Obviously, dealing with anxiety, specifically, death anxiety among aviation workers, is expected to take a toll on employee well-being and attitudes at the workplace. The findings have clearly established that anxiety is a significant determinant of both adaptive and task performance among employees. This may create a challenge for both the operations of the business and the customers they serve at large. In the era of this pandemic where aviation employees are increasingly being confronted with a lot of worry and fear about contracting or even dying from the virus due to their interactions with travelers, employers must also strive to provide appropriate psychological and emotional guidance for all employees. Additionally, employees should be trained and retrained in adhering to the needed safety protocols as spelt out by the WHO and provide them with the necessary PPEs at all times to enable them exhibit positive behaviour at the workplace. It is also important to highlight that young employees are the most vulnerable to anxiety due to their inexperience; hence, in this study, where most respondents fall within the age range of 25–35 years, managers should integrate death-related educational modules that can inform young employees to proactively handle all forms of negative thoughts that might appear in their minds.

Undebatably, COVID-19 offers a rare situation, where death stares in the eyes of most people every day due to the daily updates on the rise in infections and death tolls on different social media platforms, the television, radio, as well as the print media. As dangerous as the virus may seem to appear, adherence to the health and safety protocols and the access, availability, and use of PPEs remain a vital defensive mechanism for all. Regardless, the subject of anxiety will always be a subject of discussion, and the thought of death and how it may likely influence human behaviours can never be ruled out as demonstrated in this study. Maintaining a healthy and safer work environment in an era of a pandemic should therefore involve a collaborative effort from both the employer and the employee for a satisfactory outcome. As the employer provides the needed PPEs and training on its usage, including orienting employees on the health and safety protocols, the employees must also make efforts to adhere to these protocols and use the PPEs accordingly. It is important to reiterate that fighting anxiety among workers in an era of a pandemic will definitely take time, and the thought of death will forever remain in the minds of employees; however, the aviation sector, as well other organizations facing similar threats of COVID-19, can still control the spread and prevention of the virus through strict adherence of all safety protocols, as indicated by the WHO.

### 5.1. Strengths and Limitations

One of the major strengths of this study is the cross-sectional nature employed by the study. The large sample size and sampling technique adopted is also expected to represent the population of employees’ working in the aviation sector in Ghana. The proxies used to measure work behaviour and anxiety is a multi-dimensional construct and represents the issues that this study seeks to address. Again, the study’s methodology design and conceptual framework were built on a strong foundation that is supported by empirical studies and theories. The structural equation modelling method of data analysis as used in this study is considered as a very robust statistical tool; hence, data collected for the study have been subjected to strong preliminary assumptions and estimations before drawing conclusions on the results. This is a major strength that confirms the validity and reliability of the results. Irrespective of these strengths, the study encountered some limitations. All constructs measuring specific variables used in the study were based on self-report measures rather than the use of clinical or diagnostic evaluation. Data collection took longer than necessary, and unfortunately, most respondents were reluctant to participate or give accurate responses due to the fear of the research implications on their job.

### 5.2. Future Studies

Based on the context of this study, future research may profit through conducting a study of such nature on emerging economies to shed more light on the research issue. Other statistical analyses can also be utilized to ascertain the efficacy of the relationships explored in this study.

## Figures and Tables

**Table 1:** Description of respondents.

Gender	Frequency	Percentage
Male	278	43.0
Female	368	57.0
Total	646	100.0
Age		
Under 25 years	223	34.5
26–35 years	204	31.6
36–45 years	123	19.0
46–60 years	96	14.9
Total	646	100.0
Marrital Status	Frequency	Percent
Married	234	36.2
Unmarried	336	52.1
Divorced	57	8.8
Widow/Widower	19	2.9
Total	646	100.0
Number of Dependence		
None	189	29.3
1–3	321	49.7
4–7	89	13.7
8–10	47	7.3
Total	646	100.0
Work Experience		
Under 1 year	49	7.6
1–2 years	116	18.0
3–5 years	187	29.0
6–10 years	196	30.3
over 10 years	98	15.1
Total	646	100.0
Customer/Human Engagements at Work		
Not often	79	12.3
Often	333	51.5
Very often	234	36.2
Total	646	100.0
Frontline Workers		
Yes	472	73.1
No	98	15.2
It depends	76	11.7
Total	646	100.0

**Table 2:** Results for descriptive and reliability analysis.

Exogenous Variables	Items	Mean	SDV	α	SK	KT	KMO	BTS.
Accessibility of PPEs (APPE)	14	3.227	0.473	0.779	−1.358	2.210	0.792	0.000
1. PPEs Availability (AAP)	5	3.121	0.393	0.796	−1.002	1.995		
2.Training and Understanding PPEs Use (TUP)	4	3.001	0.405	0.735	−0.989	1.298		
3. PPEs Disposal (DSP)	5	2.224	0.442	0.808	−1.217	2.012		
Endogenous Variables								
Work Behaviours	12	2.928	0.477	0.766	−0.927	1.484	0.822	0.000
Stress Performance (SAFPEF)	4	3.001	0.362	0.703	−0.699	1.199		
Interpersonal Performance	4	2.999	0.514	0.819	−0.805	1.222		
Task Performance (TASPEF)	4	3.209	0.503	0.777	−0.777	1.203		
Mediator—Anxiety (ANXI)	10	4.258	0.339	0.915	−1.403	2.280	0.867	0.000
Dysphoria (DSP)	3	4.001	0.123	0.939	−1.111	2.010		
Fear of Death (FDE)	4	4.245	0.231	0.895	−1.014	1.982		
Death Intrusion (DINT)	4	4.441	0.196	0.911	−0.993	1.645		

SDV = Standard deviation, α = Cronbach’s alpha, SK = skewness, KT = kurtosis, KMO = Kaiser-Meyer-Olkin Measure of Sampling Adequacy, BTS = Bartlett’s Test of Sphericity (BTS).

**Table 3:** Results of convergence validity of scales.

Accessibility of PPEs (APPE)—Exogenous	AVE	CR
1. PPE Availability (AAP)	0.687	0.948
2. Training and Understanding PPE Use (TUP)	0.767	0.887
3. PPE Disposal (DSP)	0.679	0.921
Work Behaviours		
Stress Performance (STPEF)	0.689	0.956
Interpersonal Performance (INTPEF)	0.703	0.804
Task Performance (TASPEF)	0.753	0.860
Anxiety—ANXI		
Dysphoria (DSP)	0.678	0.815
Fear of Death (FDE)	0.633	0.772
Death Intrusion (DINT)	0.718	0.818

**Table 4:** Results of discriminant validity.

APPE	AAP	TUP	DSP
AAP	**(0.828)**		
TUP	0.521 ***	**(0.875)**	
DSP	0.477 ***	0.591 ***	**(0.824)**
Work Behaviours	STPEF	INTPEF	TASPEF
STPEF	(0.830)		
INTPEF	0.442 ***	(0.838)	
TASPEF	0.539 ***	0.456 ***	(0.868)
ANXI	DSP	FDE	DINT
DSP	(0.823)		
FDE	0.633 ***	(0.796)	
DINT	0.568 ***	0.544 ***	**(0.847)**

*** Significant at 95%, note: figures in bold represent the square root of the AVE.

**Table 5:** Results of goodness-of-fit for SEM.

Fit Indices	Results	Criteria
$\chi^{2}/df$	4.001	<5
GFI	0.889	>0.80
SRMR	0.027	<0.08
RMSEA	0.041	<0.08
NFI	0.875	>0.80
CFI	0.967	>0.80
TLI	0.911	>0.80

($\chi^{2}/df$ = chi-square, GFI = Goodness-of-fit index, SRMR = Standardised Root Mean Residual, RMSEA = The Root Mean Square Error of Approximation, NFI = Normed fit index, CFI = comparative fit index, TLI = Tucker-Lewis Index).

**Table 6:** Path estimates of SEM.

Paths	Standardized Estimates	S.E.	C.R.	*p*
STPEF <--- ANXI	0.289 ***	0.099	2.919	0.001
INTPEF <--- ANXI	0.165	0.068	2.426	0.079
TASPEF <--- ANXI	0.137 ***	0.101	1.356	0.003
STPEF <--- APPE	0.113 ***	0.109	1.036	0.000
INTPEF <--- APPE	0.197 ***	0.124	1.589	0.033
TASPEF <--- APPE	0.221 ***	0.118	1.873	0.001
ANXI <--- APPE	0.331 ***	0.087	3.805	0.000

*** Significant at 95%.

**Table 7:** Mediation analysis.

Mediator	Path Estimates	Estimate	*p*-Value
ANXI	ANXI <--- APPE	0.331	0.000
	STPEF <--- ANXI	0.289	0.000
Direct Effect	STPEF <--- APPE	0.113	0.000
Indirect Effects	STPEF <--- ANXI <--- APPE	0.010	0.013
Total Effects		0.213	0.000
ANXI	INTPEF <--- ANXI	0.165	
	ANXI <--- APPE	0.331	
Direct Effect	INTPEF <--- APPE	0.173	0.000
Indirect Effects	INTPEF <--- ANXI <--- APPE	0.055	0.137
Total Effect		0.228	0.085
ANXI	TASPEF <--- ANXI	0.137	0.000
	ANXI <--- APPE	0.331	0.000
Direct Effect	TASPEF <--- APPE	0.221	0.000
Indirect Effects	TASPEF <--- ANXI <--- APPE	0.045	0.007
Total Effect		0.266	0.085
Mediator—DSP	Path Estimates	Estimate	*p*-Value
Direct Effect	STPEF <--- APPE	0.113	0.000
Indirect Effects	STPEF <--- DSP <--- APPE	0.007	0.000
Total Effects		0.120	0.000
Direct Effect	INTPEF <--- APPE	0.173	0.000
Indirect Effects	INTPEF <--- DSP <--- APPE	0.039	0.091
Total Effect		0.212	0.106
Direct Effect	TASPEF <--- APPE	0.221	0.000
Indirect Effects	TASPEF <--- DSP <--- APPE	0.031	0.041
Total Effect		0.252	0.027
Mediator—FDE	Path Estimates	Estimate	*p*-Value
Direct Effect	STPEF <--- APPE	0.113	0.000
Indirect Effects	STPEF <--- FDE <--- APPE	0.014	0.000
Total Effects		0.127	0.000
Direct Effect	INTPEF <--- APPE	0.173	0.000
Indirect Effects	INTPEF <--- FDE <--- APPE	0.021	0.000
Total Effect		0.194	0.000
Direct Effect	TASPEF <--- APPE	0.221	0.000
Indirect Effects	TASPEF <--- FDE <--- APPE	0.022	0.000
Total Effect		0.243	0.000
Mediator—DINT	Path Estimates	Estimate	*p*-Value
Direct Effect	STPEF <--- APPE	0.113	0.000
Indirect Effects	STPEF <--- DINT <--- APPE	0.018	0.000
Total Effects		0.131	0.000
Direct Effect	INTPEF <--- APPE	0.173	0.000
Indirect Effects	INTPEF <--- DINT <--- APPE	0.042	0.001
Total Effect		0.215	0.001
Direct Effect	TASPEF <--- APPE	0.221	0.000
Indirect Effects	TASPEF <--- DINT <--- APPE	0.038	0.000
Total Effects		0.259	0.000

**DINT**—Death intrusion, **FDE**—Fear of death, **DSP**—Dysphoria.

## Data Availability

The data presented in this study are available on request from the corresponding author.

## Abbreviations

Abbreviation	Explanation
APPE	The construct for measuring Use and Accessibility of PPEs
AAP	The scale for measuring PPEs Availability
APE 1–6	Questionnaire items for measuring PPEs Availability
TUP	The scale measuring Training and Understanding PPEs Use
TP 2–5	Questionnaire items for measuring Training and Understanding PPEs Use
DPP	The scale measuring PPEs Disposal
DP 1–4	Questionnaire items for measuring PPEs Disposal
STPEF	The scale for measuring Stress Performance
SPEF 1–3	Questionnaire items for measuring Stress Performance
INTPEF	The scale for measuring Interpersonal Performance
ITP 1–5	Questionnaire items for measuring Interpersonal Performance
TASPEF	The scale for measuring Task Performance
TSP 1–3	Questionnaire items for measuring Task Performance
ANXI	The Construct for measuring Anxiety of the Fear of Death
DSP	The scale measuring Dysphoria
DS3-5	Questionnaire items for measuring Dysphoria
FDE	The scale measuring Fear of Death
FD 1–4	Questionnaire items for measuring Fear of Death
DINT	The scale measuring Death Intrusion
DIN 2–4	Questionnaire items for measuring Death Intrusion
